# Identification of Hepatotropic Viruses from Plasma Using Deep Sequencing: A Next Generation Diagnostic Tool

**DOI:** 10.1371/journal.pone.0060595

**Published:** 2013-04-17

**Authors:** John Law, Juan Jovel, Jordan Patterson, Glenn Ford, Sandra O’keefe, Weiwei Wang, Bo Meng, Deyong Song, Yong Zhang, Zhijian Tian, Shawn T. Wasilenko, Mandana Rahbari, Troy Mitchell, Tracy Jordan, Eric Carpenter, Andrew L. Mason, Gane Ka-Shu Wong

**Affiliations:** 1 Department of Medicine, University of Alberta, Edmonton, Alberta, Canada; 2 Department of Biological Sciences, University of Alberta, Edmonton, Alberta, Canada; 3 BGI-Shenzhen, Shenzhen, Guangdong, China; Tel Aviv University, Israel

## Abstract

We conducted an unbiased metagenomics survey using plasma from patients with chronic hepatitis B, chronic hepatitis C, autoimmune hepatitis (AIH), non-alcoholic steatohepatitis (NASH), and patients without liver disease (control). RNA and DNA libraries were sequenced from plasma filtrates enriched in viral particles to catalog virus populations. Hepatitis viruses were readily detected at high coverage in patients with chronic viral hepatitis B and C, but only a limited number of sequences resembling other viruses were found. The exception was a library from a patient diagnosed with hepatitis C virus (HCV) infection that contained multiple sequences matching GB virus C (GBV-C). Abundant GBV-C reads were also found in plasma from patients with AIH, whereas Torque teno virus (TTV) was found at high frequency in samples from patients with AIH and NASH. After taxonomic classification of sequences by BLASTn, a substantial fraction in each library, ranging from 35% to 76%, remained unclassified. These unknown sequences were assembled into scaffolds along with virus, phage and endogenous retrovirus sequences and then analyzed by BLASTx against the non-redundant protein database. Nearly the full genome of a heretofore-unknown circovirus was assembled and many scaffolds that encoded proteins with similarity to plant, insect and mammalian viruses. The presence of this novel circovirus was confirmed by PCR. BLASTx also identified many polypeptides resembling nucleo-cytoplasmic large DNA viruses (NCLDV) proteins. We re-evaluated these alignments with a profile hidden Markov method, HHblits, and observed inconsistencies in the target proteins reported by the different algorithms. This suggests that sequence alignments are insufficient to identify NCLDV proteins, especially when these alignments are only to small portions of the target protein. Nevertheless, we have now established a reliable protocol for the identification of viruses in plasma that can also be adapted to other patient samples such as urine, bile, saliva and other body fluids.

## Introduction

Traditionally, techniques such as cell culture, electron microscopy and serology have been instrumental in discovering unknown viruses. These procedures led to the identification of the viruses causing hepatitis A and B [1], [2], acquired immune deficiency syndrome [3], and acute respiratory syndrome [4]. Classical microbiology and molecular biology methods also led to the somewhat serendipitous discovery of Acanthamoeba polyphaga mimivirus [5], a member of the nucleo-cytoplasmic large DNA viruses (NCLDV) that has been proposed to constitute a fourth domain of life [6]. In contrast, the discovery of hepatitis C virus (HCV) required a more targeted approach employing screening of bacteriophage display libraries with immune serum, thus heralding a new era of virus discovery [7]. More recently, an unbiased approach has been used to identify viruses by DNAse treatment of serum, followed by amplification and sequencing of the nucleic acids protected in virions [8]. Using this method, not only was hepatitis B virus (HBV) identified in the serum of an infant with acute hepatitis B, but so too were two species of bovine parvoviruses, possibly because bovine serum was used to dilute the experimental samples [8].

With the advent of next generation sequencing (NGS) [9], the application of metagenomics to virus discovery has become more feasible. For instance, a metagenomic survey of the microflora of hives and royal jelly led to the identification of Israeli acute paralysis virus in honeybee colonies exhibiting collapse disorder [10]. A similar approach was used to study the etiology of a human disease by sequencing cDNA from a Merkel cell cancer, where a viral transcript corresponding to a novel polyomavirus was identified and referred to as the Merkel cell polyomavirus [11]. A metagenomic survey was also employed to examine infected tissues from three patients that had received liver or kidney transplantation from the same donor [12]. BLASTx alignments of 94,000 sequences yielded 14 hits that shared high similarity to lymphocytic choriomeningitis virus. The infectivity of the virus was subsequently confirmed combining cell culture, electron microscopy, and immunochemical assays [12]. Additional contributions of metagenomics to clinical virology include the identification of a bunyavirus in patients with thrombocytopenia and leukopenia syndrome [13]. Characterizations of the human gut virome have revealed the diversity and predominance of bacteriophages in this environment [14], [15]. Recently, NGS was employed to determine the complexity of the human virome in febrile children with acute diarrhea [16], [17]. In addition, novel viruses from human and animal samples have been discovered, although their pathogenicity remains to be ascertained in most cases [18]–[23].

NGS metagenomics has also been used in several environmental studies to discover new species of viruses [24], [25]. These studies estimated an abundance of 10^7^ viruses per milliliter of water in marine environments [24]. Others have identified a series of giant viruses in addition to the Acanthamoeba polyphaga mimivirus [26] and characterized virophages as small viruses that infect larger viruses [27], [28].

Detection of viruses in patients suffering from chronic disease adds an additional challenge because viral burden may be diminished with disease progression. Nevertheless, NGS technologies are evolving at a rapid pace to discover unknown agents and an accompanying body of free software is currently available for data analysis. Accordingly, our goal was to provide a detailed method for the construction of unbiased metagenomic libraries from body fluids, as well as a thorough bioinformatics pipeline for the analysis of sequencing data. In this report, we describe a viral metagenomic survey in plasma from patients affected by several hepatic disorders. DNA and RNA libraries enriched for viruses were sequenced using the Illumina GAII platform. Over 300 million high quality sequences were analyzed, enabling the identification of a series of viruses previously diagnosed by conventional assays as well as of several novel viral sequences.

## Materials and Methods

### Ethics Statement

Plasma samples were obtained from patients who read and signed the consent form according to protocols that were reviewed and approved by the Human Research Ethics Boards of the University of Alberta.

### Samples and Preparation of Nucleic Acids

Viral particles were enriched from 4–8 ml of plasma, from either single patients or pooled samples of several patients with the same diagnosis, depending upon sample availability. Plasma was spun down at 2,655×g, for 5 min, at 4°C. The supernatant volume was brought up to 20 ml with TN buffer (0.1 M Tris, pH 7.6; 0.1 M NaCl), and the suspension run through a 0.2 µm MidJet column (GE Healthcare) at 4°C, to filter out bacterial and human cells. Samples were concentrated in a final volume of 8 ml by tangential flow filtration, using 300 KDa MidJet columns at 4°C. Half of the concentrated suspension was kept at −85°C. The remaining 4 ml were treated with 50 U/ml Benzonase (Sigma) in 2 mM MgCl_2_, at 21°C for 1.5 h. RNA was extracted from 2 ml of concentrated suspension with the QIAamp Viral RNA Mini Kit (Qiagen) according to manufacturer’s instructions. As multiple columns were needed to process the 2 ml sample, all 60 µl eluates were pooled. The RNA was then concentrated and treated with DNase I in a single column, using the Qiagen RNeasy Microkit as recommended in the RNA Cleanup protocol, except that the DNase I amount was raised to 81 U. RNA was finally eluted in 25 µl RNase-free water. DNA was isolated from the other 2 ml fraction of concentrated suspension, using the DNeasy Blood and Tissue Kit (Qiagen) as described in the Purification from Animal Blood or Cells protocol, including treatment with RNase A. DNA was finally concentrated and eluted from all columns by sequentially passaging the same 80 µl buffer AE aliquot (∼40 µl were recovered from the last column).

### Construction and Sequencing of Metagenomics Libraries

Seven RNA libraries (aihP01, hbvP02, hcvP02, hcvP03, hcvP05, nshP01, norP01) and seven DNA libraries (aihP01D, hbvP02D, hcvP02D, hcvP03D, hcvP05D, nshP01D, norP01D) were constructed from patients with autoimmune hepatitis (AIH), hepatitis B virus (HBV) chronic infection, hepatitis C virus (HCV) chronic infection, non-alcoholic steatohepatitis (NASH), and healthy subjects (NOR). Plasma from four patients attending an outpatient surgery ward without any known diagnosis of liver disease were pooled and used as control (sample NORP01). Plasma from three patients affected by AIH or NASH were also pooled in samples AIHP01 and NSHP01, respectively. The rest of samples derived from individual patients.

For RNA library preparation, first strand cDNA was synthesized from half of the RNA amount obtained during purification (usually below detection limit by spectrophotometry) in the presence of 3 µg of random hexamers (Invitrogen), 0.5 mM dNTPs, 40 U of RNaseOUT (Invitrogen), 100 U of Reverse Transcriptase SuperScript II (Invitrogen), and 1X first strand buffer (provided by the manufacturer), for 50 min at 42°C. For second strand synthesis, the first strand reaction was supplemented with 50 mM Tris pH 7.8; 5 mM MgCl_2_, 0.3 mM dNTPs, 1 mM DTT, 2 U of RNase H and 50 U of DNA pol I, and further incubated at 16°C for 2.5 h. For DNA library preparation, purified DNA was sheared in a Covaris S2 instrument, using 6×16 mm AFA fiber snap-cap microtubes (Covaris), and recommended conditions to generate a target peak of 200 nt (duty cycle: 10%, intensity: 5, cycles per burst: 200, treatment time: 180 sec, water bath temperature: 7°C).

From this point on, RNA and DNA libraries were processed with a common protocol. DNA protruding ends were blunted with the NEBNext End Repair Module (NEB). A 3′ –dA overhang was added with the NEBNext dA-Tailing Module (NEB). Finally, ligation-competent DNA molecules were ligated to paired-end (PE) adapters harboring 3′ –dT overhang (Illumina) using the NEBNext Quick Ligation Module (NEB), all according to protocols provided by manufacturers. Samples were cleaned up using the QIAquick PCR purification kit (Qiagen) after end repair or dA-tailing and with the MinElute PCR purification kit (Qiagen) after ligation. Elution volumes were adjusted as required for the next reaction. After ligation, samples were fractionated in a 2% agarose gel and a thin slice at ∼300 nt was excised with 4.0 mm×1.0 mm GeneCatcher disposable gel excision tips (Gel Company) and the DNA was recovered with the QIAquick Gel Extraction Kit (Qiagen).

Libraries were then amplified with 50 U of PfxIII polymerase (Invitrogen) in the presence of 1X reaction buffer (provided by the manufacturer), 1 µM primers [29] and 0.25 mM dNTPs. After 18 PCR cycles, amplification was titrated in a 2100 Bioanalyzer (Agilent) and additional PCR cycles were added as needed. When required, amplified adapter dimmers were removed with Agencourt AMPure magnetic beads (Beckmann Coulter). Libraries were size selected on a 2% agarose gel. All libraries were sequenced at BGI-Shenzhen (China), on a Genome Analyzer II instrument (Illumina). Sequencing data is available from the SRA repository under the following accession number: SRA054231.

### Data Analysis

Low quality bases (quality score “B”) were trimmed from the 3′ end of each read end, and the remaining sequence was kept only when its high quality 5′ moiety was longer than 29 nt. Primer/adapter sequences and low complexity regions were also trimmed out. SOAPaligner [30] was used to remove mitochondrial and ribosomal RNAs, using the default parameters. RepeatMasker [31] was then used with a database containing simple repeats, ribosomal and mitochondrial sequences, to further filter the reads with higher sensitivity. The remaining sequences were considered clean reads. To minimize CPU time, clean reads were aligned to the human, bacteria, and virus databases from NCBI, using SOAPaligner with the default parameters. Remaining read ends were aligned with standalone BLASTn against the above-described databases. An E-value of 1e–05 was used as the cutoff. In the specific case of the human database, 80% coverage and 80% identity were also required. When a query hit more than one taxon, hits were sorted by E-value and additional taxa were kept if their hits were within two nucleotides of the top hit. For example, if a query hit the human database with 55 identical nucleotides and the virus database with 53 identical nucleotides, the query sequence was then assigned to the ambiguous category human-virus (HV). Additionally, the taxonomy assigned to each end of a pair was compared and the ambiguity solved when both ends intersected the same taxon. For instance, for a given pair, if one end is classified as HV and its counterpart as H, this pair was reclassified as H-H. Read ends that could not be classified were binned as unknown.

Non-human and non-bacteria (unknown, viral, phage, human endogenous retroviruses [HERV] and ambiguous) read ends were subjected to *de novo* assembly with the SOAP*denovo*-Trans software (http://soap.genomics.org.cn/SOAPdenovo-Trans.html), using the default parameters. We chose the transcriptome assembler SOAP*denovo*-Trans, instead of the older genome assembler SOAP*denovo*, because it takes into account the problem of uneven coverage, which is present in both transcriptomic and metagenomic libraries. Assembled scaffolds were aligned with BLASTx against the ‘nr’ taxonomy databases, including archaea, bacteria, HERV, fungus, plant, human, invertebrate, mammal, phage, protist, vertebrate and virus entries. As before, an E-value cutoff of 1e-05 was used and the top hit was reported. In the specific case of phycodnavirus and mimivirus top hits, some scaffolds were re-analyzed with the HHblits algorithm, using the most recent version of the UniProt20 database [32]. HHblits utilizes profile hidden Markov models to represent both query and database sequences; these profiles are then aligned using HHsearch [33].

### Verification of Virus Presence by PCR

Total RNA was isolated from 140 µl of plasma using the QIAamp viral RNA kit (Qiagen), followed by random-primed cDNA synthesis using 1 µg of RNA and SuperScriptII, as described above. PCR was performed using Platinum Taq High Fidelity polymerase (Invitrogen) in the presence of 0.5 mM HCV-specific primers (HCV-1: CTCCCCTGTGAGGAACTACTGTCT, HCV-2: CTCGCAAGCACCCTATCAGGCAG), which anneals to conserved motifs in the 5′ untranslated region of the HCV genome. Likewise, DNA was used as template to detect TTV with the following primers TTV-1: GTGGGACTTTCACTTGTCGGTGTC, TTV-2: GACAAATGGCAAGAAGATAAAGGCC. A fragment of Scaffold2306 was amplified using primers Circo1: TGTCATAGGCAAACCTAGCACCGT and Circo2: TATGTGGACCCATCCGAAAGCCTT. PCR conditions were as recommended by the polymerase manufacturer. The HCV and TTV primers bind to conserved sequences in many strains of the corresponding virus, while circovirus primers were deduced from the sequence of Scaffold2306 (sequence available upon request).

## Results

### Libraries and Bioinformatics Pipeline

We defined the complexity of viruses present in the plasma of patients with chronic liver disease, using metagenomics. We chose plasma to filter out bacterial and human cells with the consequent enrichment of virus particles. In the context of virus identification and discovery using NGS, the removal of bacterial and human cells is highly desirable because their ribosomal and mitochondrial RNA fractions are highly represented contaminants that often hinder detection of viral nucleic acids. Our viral preparations were made from pooled plasma samples and split into two fractions for the preparation of RNA and DNA libraries using a protocol that minimizes handling of samples to preserve virome diversity. Plasma samples were obtained from patients with autoimmune hepatitis (AIH), chronic hepatitis B virus (HBV) infection, chronic hepatitis C virus (HCV) infection, non-alcoholic steatohepatitis (NASH), and healthy subjects (NOR); each RNA library was named by diagnosis (*e.g.* aihP01) and a suffix ‘D’ was added for each DNA library (*e.g.* aihP01D).

**Figure 1:**
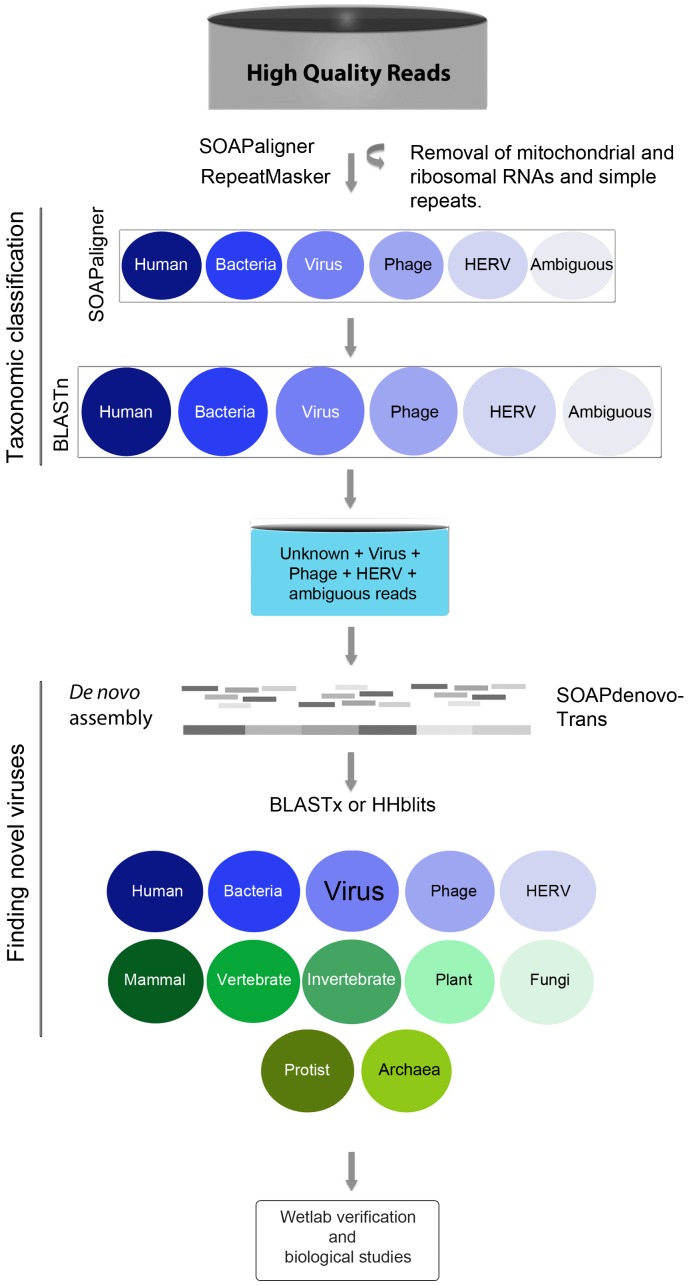
Schematic of bioinformatics pipeline used for processing of NGS libraries. High quality reads, excluding ribosomal and mitochondrial sequences, were aligned against the taxonomy databases of NCBI using BLASTn (taxonomic classification). Unclassified or ambiguously classified reads, together with virus, phage, and HERV sequences were assembled into scaffolds. Scaffolds were used to query the non-redundant protein database of NCBI using BLASTx to identify viral proteins with similarity to predicted polypeptides in our scaffolds (finding novel viruses). Given the large genomes of NCLDVs, hits to this class of viruses were reanalyzed with the profile hidden Markov model-based algorithm HHblits. PCR and Sanger sequencing were used to confirm the presence of novel viral-like sequences in our samples.

**Figure 2:**
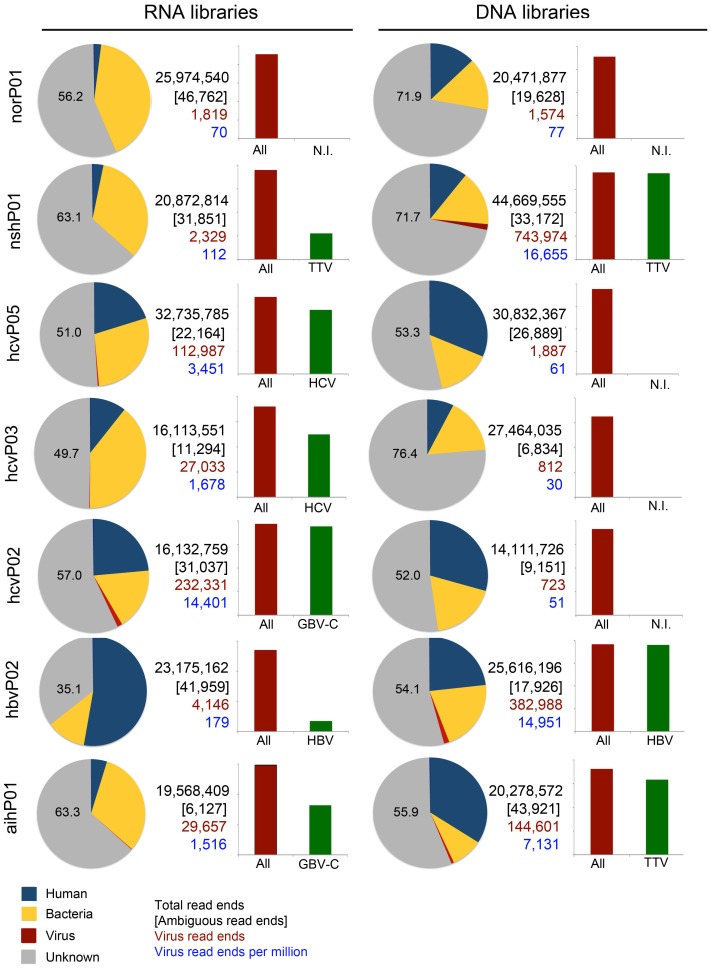
Viral read ends represent only a small fraction of libraries from plasma. Pie charts: Classification of reads from each library into human, bacteria, virus, and unknown categories (HERV and phage sequences are not included as they were found at low frequency, not visible at the scale of the pie charts). The number of clean reads (high-quality sequences excluding ribosomal, mitochondrial, and low complexity sequences; in black), ambiguous reads (in brackets), viral reads (in red), and the density of viral reads per million (in blue) are indicated. Bar graphs: Red bars depict total number of viral reads (normalized to 100%) and green bars represent the percentage thereof that corresponds to the most abundant virus in each library (N.I. not identified; indicates that no predominant virus was identified during BLASTn alignments).

Sequences were analyzed using an in-house bioinformatics pipeline depicted in Figure 1 (see Materials and Methods). We performed a taxonomic classification of reads into human, bacteria, phage, human endogenous retroviruses (HERV), viruses, and unknown categories (Supplemental Tables S1–S14). A significant fraction of reads in each library could not be unambiguously assigned to a definitive category; these were therefore included into several ambiguous categories describing the combinations of taxa that were matched (Supplemental Tables S1–S14; Figure 2, in brackets). Notably, the vast majority of reads in each library did not bear resemblance to any of the taxa available in the NCBI databases; these were assigned to the category “unknown” (Figure 2; Supplemental Tables S1–S14). They represent a pool of sequences that can potentially be assembled into new genomes or segments thereof. Although our filtration procedure was intended for enrichment of virus particles, some human, bacterial and phage nucleic acids escape tangential flow filtration – most likely when present in a cell-free form. However, our focus was directed to the analyses of virus populations and virus discovery.

### Complexity of Viral Sequences in Plasma Samples

Using viral preparations, the ratio of known viral to non-viral read ends was 1∶229 (Figure 2). The density of viral read ends was highly variable and ranged from 70 to 14,401 read ends/million for the RNA libraries and 30 to 16,655 read ends/million for the DNA libraries. In most cases where a relatively high density of viral sequences was found, there was a single predominant virus (Supplemental Tables S15–S28). For instance, in all libraries with more than 100,000 viral sequences, a single virus and its quasi-species accounted for more than 75% of viral sequence abundance (Figure 2; bar graphs). It is notable that DNA viruses were detected in RNA as well as DNA libraries, such as HBV and Torque teno virus (TTV). However, it is unknown whether this finding represents the detection of viral transcripts or the inability to eradicate DNA in the RNA libraries. The relative abundance of HBV- and TTV-like sequences in RNA libraries compared to DNA libraries was 0.20% and 0.13%, respectively (HBV in sample hbvP02∶30 vs 14,786 read ends/million; TTV in sample nshP01∶22 vs 16,483 read ends/million). To a lesser extent (about one read per million), we also detected sequences resembling RNA viruses in our DNA libraries (Supplemental Tables S15–S28). This may represent alignment inaccuracies or stretches of unknown DNA viruses that resemble RNA viruses. Retroviruses constitute a special case, as they are RNA viruses that retro-transcribe into DNA, and integrate into human chromosomes. On occasion, the proviral genome is found in the viral particle and therefore retroviral sequences may be observed in both DNA and RNA libraries.

**Figure 3:**
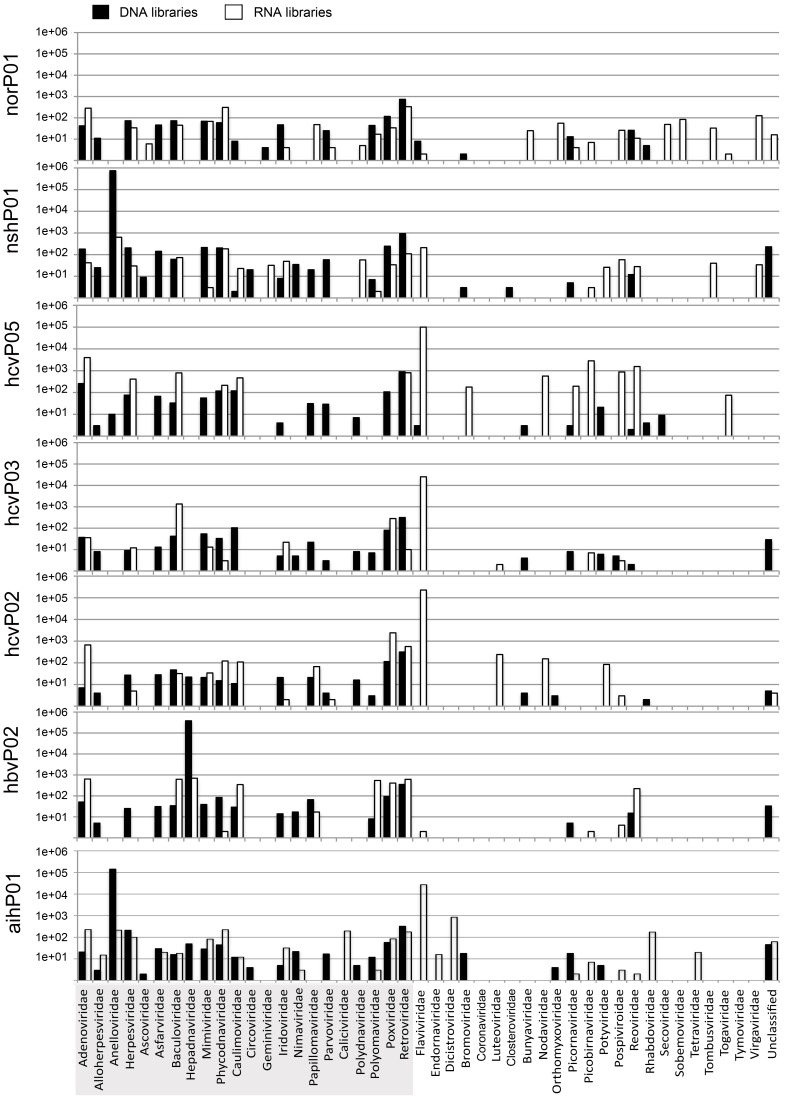
Classification of viral reads into families reveals high diversity of viruses in plasma samples. Reads were classified into families according to the taxonomy databases of NCBI. Solid bars represent DNA libraries and open bars represent RNA libraries. Data is presented in log10 scale. The names of the DNA viral families are shadowed (bottom).

Viruses from diverse families were found within each library (Figure 3). Anelloviruses, small circular single-stranded DNA viruses, were predominant in AIH and NASH DNA libraries, but poorly represented in the rest of samples. As expected, hepadnaviruses were found in a patient diagnosed with HBV and we found abundant flaviviruses in patients diagnosed with HCV. Of note, the predominant virus detected in library hcvP02 was not HCV, but GB virus C (GBV-C), a flavivirus that was also detected in the AIH library (Figures 2 and 3). In general, the expected hepatitis viruses were detected in plasma from patients with the appropriate clinical diagnosis, but in some cases the deep sequencing provided additional information. Few hits to viral sequences were found in the libraries norP01 and norP01D used as a negative comparison group for hepatotropic viruses, (ratio to non-viral reads 1∶13,689; Figure 2).

### Predominant Viruses Detected in Libraries

**Figure 4:**
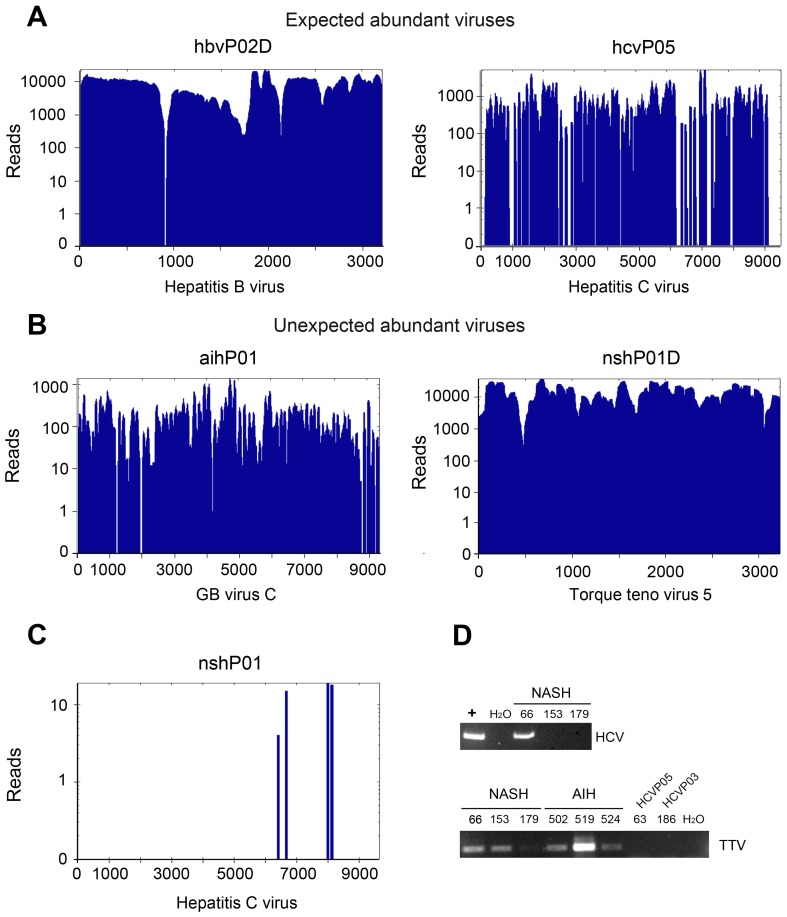
NGS identified several classes of viruses that would otherwise remain undetected. Panels A to C show the mapping of reads along the indicated viral genome, (**A**) *Expected abundant viruses* were recovered at high and uniform coverage as illustrated for HBV (NC_003977.1) in library hbvP02D and HCV genotype 2 (NC_009823.1) in library hcvP05. (**B**) *Unexpected abundant viruses* were also found at high and uniform coverage, as illustrated for GBV-C/Hepatitis G virus (NC_001710.1) in library aihP01 and TTV 5 isolate TCHN-C1 (AF345523.1) in library nshP01D. (**C**) A few read ends mapping to HCV genotype 1 (NC_004102.1) were found in library nshP01, which initially tested negative for HCV in serological tests. (**D**) HCV was PCR amplified in one of the three samples pooled for construction of library nshP01 (upper panel). Additionally, TTV was found in samples NSH and AIH, but not in samples from patients affected by chronic hepatitis C (HCVP03 and HCVP05).

Nearly complete viral genomes were detected at high coverage in the libraries from patients with chronic viral hepatitis. For example, the DNA genome of HBV was covered several thousand times by reads in library hbvP02D (Figure 4A). A single locus was not covered in the HBV sequence that we used as our reference (NC_003977.1), possibly related to polymorphisms in different genotypes. Relatively good coverage was also found in HCV libraries hcvP05 (Figure 4A) and hcvP03 (data not shown). We also observed nearly full coverage of GBV-C in sample aihP01 (Figure 4B) and hcvP02 (not shown) as well as TTV in samples nshP01D (Figure 4B) and aihP01D (data not shown). Since the aihP01 and nshP01 samples contained pooled plasma from three patients, it was not possible to ascertain which of these patients were infected by TTV or GBV-C. We replicated the AIH libraries with serum from a different patient, but neither TTV nor GBV-C were detected (data not shown) suggesting no relationship between TTV and/or GBV-C and disease. However, detection of such viruses in our libraries illustrates the sensitivity of our approach to conduct unbiased diagnoses of viral agents in plasma.

Of note, we observed 128 sequences that aligned to the HCV genotype 1 in a library derived from pooled plasma of three patients with NASH (patients 66, 153 and 179; Figure 4C). This was unexpected because patients suffering from NASH are usually tested for the presence of HCV prior to diagnosis [34]. Therefore, we conducted RT-PCR on RNA from each patient’s plasma in duplicate, using HCV-specific primers. We found that the plasma from one patient in the pool used for library nashP01 was positive for HCV RNA (Figure 4D). Similarly, we were able to confirm the presence of TTV DNA in patients affected by NASH and AIH, but not in those affected by hepatitis C (Figure 4B). We detected ten reads mapping to TTV in library hcvP05D, out of ∼31 million sequences, which explains our inability to detect this virus in PCR experiments.

### Virus Discovery of Novel Agents in Plasma Samples

Reads that were not classified as human or bacteria were subjected to *de novo* assembly using the SOAP*denovo*-Trans algorithm, and the resulting scaffolds were used to query the non-redundant protein databases. Assembled scaffolds were much more abundant in DNA libraries and most of the recognizable scaffolds hit to bacterial proteins during BLASTx runs. These data suggest that most of the reads that could not be classified during alignments with BLASTn belong to unknown bacteria. More specifically, 11% to 55% of scaffolds aligned to bacterial proteins. Nonetheless, the vast majority of scaffolds (44% to 85%) did not resemble any protein in the ‘nr’ database, which likely reflects proteins that have not yet been discovered. Assembly of viral sequences was also possible for all viruses shown in Figure 2 as the most abundant virus in each library (data not shown).

**Figure 5:**
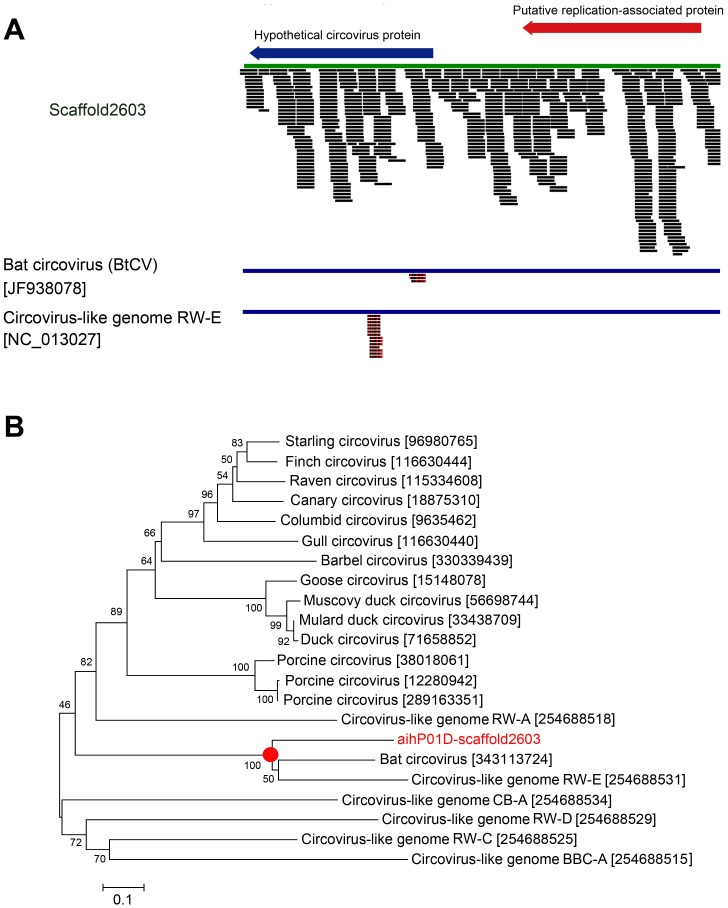
A novel circovirus genome was assembled from sequences in sample AIH. (**A**) The schematic of Scaffold2603 shows the 580 reads used for assembly and the location of the two predicted open reading frames. The few reads matched by BLASTn to the YN-BtCV-1 (AEL87784.1) and to the RW-E (NC_013023.1) circovirus reference sequences show red segments on aligned reads that represent mismatches. (**B**) Phylogenetic tree derived from the alignment of the replicase amino acid sequences from the circoviruses named on the tree branches (the GenBank identifier is also indicated). The dendrogram was calculated using the neighbor joining method and the bootstraps option (1000 replications) of the Mega 4 software (http://www.megasoftware.net/mega4/mega.html). Vertical branches are arbitrary, while the length of horizontal branches is proportional to calculated mutational distances. Numbers at nodes indicate percentage bootstrap scores.

In addition, we were able to identify a number of viruses that were not previously detected by BLASTn on the unassembled read ends (Table 1 and Supplemental Tables S29–S42). The most conspicuous were scaffolds that encoded polypeptides resembling proteins found in viruses with circular genomes and in nucleo-cytoplasmic large DNA viruses (NCLDVs). During BLASTx alignments, scaffolds from several libraries ranging in size from 600 to 2100 nt hit the replicase sequences from various circoviruses. The most striking example was Scaffold2603 in library aihP01D (Figure 5A), which was ∼2100 nt long. After refining its sequence with GapCloser for SOAP*denovo*, we found that the sequence encoded a polypeptide sharing ∼65% identity with the replicase of a Bat circovirus (BtCV; AEL87784.1; BLASTx alignments E-value 7.6e–75; 95% coverage) that was recently identified in China [35]. We then reassessed Scaffold2603 and found that it encoded an additional protein with weak similarity to a putative protein from the Circovirus-like genome RW-E [25]. Interestingly, we also assembled scaffolds resembling circoviruses in library nshP01D and, at lower frequency, in library norP01D. When the 582 original read ends from Scaffold2603 were aligned by BLASTn to the genomes of BtCV or RW-E, the two closest relatives, only a small proportion showed any similarity to the circovirus genomes (Figure 5A), supporting the genetic plasticity previously reported for this genus of viruses [25], [35], [36]. Cloning and sequencing of PCR fragments amplified from sample aihP01 verified the existence of sequences assembled into Scaffold2603. The replicase-like amino acid sequence encoded by Scaffold2603 was used to construct a phylogenetic tree of circoviral replicases and the novel protein sequence formed a separated branch with the two related circoviruses, but was divergent from the rest of the circovirus replicases (Figure 5B).

**Table 1:** Representative viral proteins identified as high scoring hits during BLASTx alignments of assembled scaffolds.

Library	Length (nt)	Average read ends depth	Unknown read ends	Target	Coverage (%)	Identities (%)	E-value
aihP01	2,716	99	3,596	Plasmopara halstedii virus A coat protein gi|301070442|	83.7	34.9	9.6E−26
aihP01D	2,110	21	582	Bat circovirus ZS/China/2011 putative replication-associated protein gi|343113724|	94.8	64.6	7.6E−75
hbvP02	–	–	–	–	–	–	–
hbvP02D	222	43	128	Orf virus PP287 gi|325073749|	63.3	84.2	4.2E−21
hcvP02	–	–	–	–	–	–	–
hcvP02D	1,426	32	611	Agrotis segetum granulovirus ORF17 gi|46309437|	53.7	34.3	8.8E−06
	297	43	170	Orf virus PP287 gi|325073749|	88.9	78.8	6.7E−32
hcvP03	–	–	–	–	–	–	–
hcvP03D	–	–	–	–	–	–	–
hcvP05	6,041	2,426	195,400	Chaetoceros sp. RNA virus 2 putative replication related protein gi|336391069|	68.1	39.0	1.3E−87
	2,419	2,829	91,271	Heterosigma akashiwo RNA virus polyprotein gi|38707889|	25.1	30.0	1.5E−52
hcvP05D	866	21	237	Trichoplusia ni ascovirus hypothetical protein TNAV2c_gp158gi|116326842|	39.7	37.8	8.7E−07
	166	13	29	African swine fever p72 protein gi|269959189|	59.3	53.9	1.2E−07
nshP01	3,054	205	8,344	Plasmopara halstedii virus A coat protein gi|301070442|	63.2	35.4	1.1E−22
nshP01D	3,230	6,441	277,377	Torque teno virus 5 Orf1 gi|13022215|	41.4	78.8	1.3E−09
	1,345	116	2,072	Bat circovirus ZS/China/2011 putative replication-associated protein gi|343113724|	98.5	58.1	3.7E−83
	1,046	39	544	Rodent stool-associated circular genome virus REP 2 gi|343196958|	80.0	63.8	5.0E−24
	634	47	396	Mosquito VEM virus SDBVL G Rep2 gi|333596697|	57.9	55.3	1.7E−31
norP01	1,335	126	2,244	Plasmopara halstedii virus A coat protein gi|301070442|	50.7	39.8	1.1E−14
norP01D	740	32	321	Bat circovirus ZS/China/2011 putative replication-associated protein gi|343113724|	50.6	52.9	2.4E−36

**Library**: id of library. **Length (nt)**: Length of scaffold in nucleotides. **Average read ends depth**: (number of read ends × length of end)/length of scaffold. **Unknown read ends**: number of read ends previously binned as unknown included in the assembly of scaffold. **Target**: target sequence ID. **Coverage (%)**: percentage of target protein included in the alignment. **Identities (%)**: percentage of amino acids identical in the query and target sequences. **E-value**: E value.

NCLDV constitute an especially challenging group of viruses to detect because they may be eliminated by size filtration and also because portions of their large genomes (>1 Mb) resemble those of higher eukaryotes [37]. NCLDV include the families *Poxviridae*, *Phycodnaviridae*, *Iridoviridae* and *Mimiviridae* [38]. We found hits to NCLDV in all libraries included in this report (Figure 3 and Supplemental Tables S29–S42). Although five conserved ortholog gene clusters have been identified within the genomes of all NCLDV [39], none of these were found in our bioinformatics analyses. Given that phycodnaviruses and mimiviruses (hereafter referred to as large viruses) have not been reported to infect humans, we re-evaluated a portion of the large virus BLASTx top hits using the HHblits algorithm [32] and the SwissProt20 database. When BLASTx and HHblits results were compared, it was observed that both algorithms aligned remarkably similar fragments of the query sequences in the corresponding top hits (Figure S1). Nonetheless, 19 out of 44 cases reported by BLASTx as large viruses proteins were re-classified as proteins from other taxa by HHblits, mainly bacterial proteins; 18 instances produced identical results (i.e. both algorithms reported the same target protein as top hit); and seven corresponded to other large viruses proteins (Supplemental Table S43). However, all were partial alignments ranging from 36 to 232 amino acids, not the entire target protein. Such divergent results are a cautionary note on the intrinsic difficulty of identifying NCLDV proteins from partial alignments. It is not possible to conclude that such sequences represent *bona fide* large viruses proteins until further experimental evidence is gathered.

## Discussion

In this study, we demonstrated the utility of NGS to analyze the complexity of DNA and RNA viral populations in the plasma of patients with chronic liver diseases. We assembled scaffolds with nucleotide sequences unrelated to all viruses reported in the databases; these sequences encoded polypeptides with similarity to previously identified viral proteins, which is consistent with the identification of novel viral agents. In libraries that contained abundant viral hits, a predominant virus was found, which usually coincided with the clinical diagnosis, such as chronic HBV and HCV infection. The exception was sample hcvP02, where the predominant species was GBV-C, another flavivirus found to replicate in primary T and B lymphocytes [40]. So far, no pathogenic properties have been attributed to GBV-C [40], [41]. Indeed, it has been suggested that GBV-C co-infection reduces the severity of HIV infection by mechanisms that remain poorly understood [40]–[43]. Although we found that one patient initially diagnosed with HCV harbored high levels of GBV-C but only small traces of HCV in plasma, it should be noted that GBV-C abundance is higher in blood than in liver while the opposite is true for HCV [42].

Neither AIH nor NASH have been linked with a specific microbial infection. However, some have speculated that AIH may be triggered by viruses [44] while others have suggested that fat may accumulate in the liver in response to virus infection [45]. Accordingly, there may be some interest in further defining the virome in plasma from these patients. The sample from patients diagnosed with AIH harbored abundant TTV and GBV-C. TTV was even more abundant in plasma from the NASH patient, but absent in patients with hepatitis B or C. Originally, TTV was isolated from a Japanese patient suffering from cryptogenic hepatitis [46], and subsequently found in serum from patients with hepatitis lacking diagnostic markers for viral infection [47]. As with GBV-C, no pathogenicity has been ascribed to TTV [34]; however, it was proposed that TTV may play an indirect role on carcinogenesis by modulating T cells immunological responses [48]. Also, increased TTV burden has been found in the plasma from immunocompromised subjects [49], [50]. Several other disorders have also been linked to anelloviruses [51], [52], but a proof for their role in pathogenesis is still lacking. Nevertheless, TTV seems to reunite many of the idealized attributes of a well-adapted virus, including persistence, high levels of replication, prevalence in the population, and high transmissibility [53].

In three of our samples (aihP01D, nshP01D and norP01D), we assembled scaffolds that contained ORFs encoding proteins similar to the replicase of a bat circovirus recently isolated in China [35] and a putative protein from another circovirus found in environmental samples [25]. Members in the family *Circoviridae* are ubiquitous, highly diverse and induce immuno-suppression in birds; at least one contributes to the development of postweaning multisystem wasting syndrome in pigs. However, no pathogenicity has been demonstrated in humans, although they are often found in human feces and plasma [18]–[22], [36], [54]. Although we found circovirus sequences in patients with NASH and AIH, their presence does not imply any linkage with liver diseases, and indeed they were also found in our control libraries.

Identification of viruses in NGS libraries relies on alignments to reference genomes or segments thereof. This method is reliable for identical or highly similar sequences but has two obvious limitations. First, viruses with novel sequences may not be detected. Second, sequences found in taxonomically divergent species may trigger spurious hits that are difficult to resolve by computational methods. In this regard, a group of viruses that deserve special attention are NCLDV that include mimiviruses and phycodnaviruses [26]. The first of the so-called giant viruses reported was Acanthamoeba polyphaga mimivirus; more recently, Giant marseillevirus, Cafeteria roenbergensis virus and Megavirus chilensis were isolated [26], [55]–[57]. The genomes of those viruses may extend up to ∼1.2 Mb and their gene complements can be as large as 1000, which by far exceeds the number of protein coding genes in many bacteria [26]. However, these large viruses have not been reported in human samples and, based on our results, we are not prepared to say that we have seen them in our samples either.

By combining viral preparations from plasma samples, deep-sequencing and computational approaches, we have shown here the identification of a series of viruses from patients affected by chronic hepatobiliary disorders. The incorporation of a viral preparation step increased the ratio of virus to human sequences (1∶229) by nearly two log-fold in comparison to a recent metagenomic study of febrile and afebrile children’s nasopharyngeal swabs and plasma samples, where approximately 1∶16,000 viral to human sequences were reported [16]. We also recovered viruses at high coverage that were previously diagnosed by traditional serological methods, while unsuspected viruses were detected in several other samples at high density. Finally, we defined a minimal threshold of abundance of viral read ends, which suffices to detect viruses present at low levels. Namely, HCV was detected in patients affected by NASH disease, at a density of approximately seven read ends per million. To make a clinical diagnosis of NASH, other liver disease agents such as HCV should be excluded and the diagnosis of NASH was therefore erroneous in the one case where HCV was detected in the metagenomics library and confirmed by PCR.

We anticipate that, due to its high sensitivity and the capability to simultaneously detect a broad spectrum of microbes, metagenomics will be extensively implemented in clinical research and diagnostics in the near future.

## Supporting Information

Figure S1 **Aligned regions of the query sequences for top hits reported by BLASTx or HHblits.** The names of the library and scaffold are included in the header of each alignment. Green background indicates identical amino acids for the two sequences included in the alignment. In all cases, the two algorithms identified the same region of the query as being similar to the target, but one algorithm would occasionally align over a slightly larger region of the query, and in doing so a different target would sometimes be chosen as top hit. Cases where BLASTx and HHblits reported different target proteins as top hits are indicated with an asterisk (*). Cases where BLASTx and HHblits reported different NCLDV proteins as top hits are indicated with two asterisks (**).
(PDF)

Supplemental Tables S1–S14 **Breakdown of sequenced read ends according to their similarity to annotated sequences.** High-quality reads were aligned to all mitochondrial and ribosomal databases available and the remnant is considered clean read ends. Clean read ends were aligned to the taxonomy databases and assigned to taxa according to E-value and percentage similarity. Reads that matched more than one taxon with similar identity (up to two divergent nucleotides) were binned as ambiguous. **Resolved Ends**: refers to read ends whose taxonomy was refined using the taxonomy of their corresponding paired end, as explained in Materials and Methods. Notice that for a particular taxon (let’s say taxon 1), ‘Resolved Ends’ could be greater than ‘Total Ends’ when read ends from a different taxon were reassigned to taxon 1. However, since it is a reclassification, the sum of read ends in all taxa should be the same for ‘Total Ends’ and ‘Resolved Ends’. Read ends that did not resemble any annotated sequence were binned as unknown.
(XLSX)

Supplemental Tables S15–S28 **Summary description of top hits to the virus database from single read ends alignments with BLASTn.** The content of each column is as follows: **Count**: number of read ends that aligned to the target sequence; **Target**: target sequence ID; **Target length (nt)**: length of target sequence in nucleotides; **Align Coverage (nt)**: length of the region covered in the target sequence by the local BLASTn alignment; **% Align Coverage**: same as before, but expressed in percentage of the target length.
(XLSX)

Supplemental Tables S29–S42 **Summary description of top hits to the virus database from scaffolds alignments with BLASTx.** The content of each column is as follows: **Scaffold**: ID of scaffold after assembly with SOAP*denovo*-Trans; **Length (nt)**: length of scaffold in nucleotides; **V Read Ends**: number of previously classified viral read ends included in the assembly of scaffold; **Amb Read Ends**: number of previously classified ambiguous read ends included in the assembly of scaffold; **U Read Ends**: number of read ends previously binned as unknown included in the assembly of scaffold; **Target**: target sequence ID; **Target AA length**: length of target sequence in amino acids; **Total** **AA Align Coverage**: Number of amino acids from the target included in the alignment; **% Align Coverage**: same as before, but expressed in percentage; **E-val**: E-value. **Identity %**: Percentage of amino acids from the alignments that are identical in the query and subject sequences.
(XLSX)

Supplemental Table S43 **Description of top hits initially identified as phycodnaviruses or mimiviruses by BLASTx and reanalyzed by HHblits.** The content of each column is as follows. **Library**: name of library; **Scaffold**: ID of scaffold after assembly with SOAP*denovo*-Trans; **Scaffold Length (nt)**: Length of scaffold in nucleotides. **Target**: Target sequence ID identified as top hit either by BLASTx or HHblits (indicated on the top row); **Target AA Length:** Length of protein targeted as top hit during alignments. **Length of hit**: Number of amino acids in the target sequence included in the alignment for the top BLASTx or HHblits hits (indicated on the top row); **Align coverage (%)**: Percentage of target length included in the alignment reported as top hit. **Identities (%)**: Percentage of aligned amino acids identical in query and target sequences. **E-val**: BLASTx or HHblits E-value (indicated on the top row). **Frame of query**: which translated reading frame was used by each algorithm. **Start of query**: coordinate of the first amino acid in the translated reading frame included in the alignment of the top hit. **End of query**: coordinate of the last amino acid in the translated reading frame included in the alignment of the top hit. **Sequence of query**: amino acid sequence of the fragment of the query included in the alignment of the top hit. **Agree?** Indicates whether or not top hits reported by BLASTx and HHblits are identical; **other NCLDV** indicates that a protein in a NCLDV different from the one reported as top hit by BLASTx was reported as top hit by HHblits.
(XLSX)
