# Supplementary material for: Identification of Hepatotropic Viruses from Plasma Using Deep Sequencing: A Next Generation Diagnostic Tool
Source: PLoS One. 2013 Apr 17;8(4):e60595. doi: 10.1371/journal.pone.0060595 (PMC3629200; doi:10.1371/journal.pone.0060595)
Supplement: Figure S1 — Aligned regions of the query sequences for top hits reported by BLASTx or HHblits. The names of the library and scaffold are included in the header of each alignment. Green background indicates identical amino acids for the two sequences included in the alignment. In all cases, the two algorithms identified the same region of the query as being similar to the target, but one algorithm would occasionally align over a slightly larger region of the query, and in doing so a different target would sometimes be chosen as top hit. Cases where BLASTx and HHblits reported different target proteins as top hits are indicated with an asterisk (*). Cases where BLASTx and HHblits reported different NCLDV proteins as top hits are indicated with two asterisks (**). (PDF) [file pone.0060595.s001.pdf]

Supplemental Figure 1

>aihP01_C2165983

```
BLASTx    ITQYIGADIGFVREWIQKMFVGNMTWENYGEVWVIDHIVPFRSFDIFNEDDLRLVWNYRNLMPI
          ||||||||||||||||||||||||||||||||||||||||||||||||||||||||||||||||
HHblits   ITQYIGADIGFVREWIQKMFVGNMTWENYGEVWVIDHIVPFRSFDIFNEDDLRLVWNYRNLMPI
```

>aihP01_C2163433*

```
BLASTx    WAEKLGIEQSAAITTVKPSGTVSQLVDSASGIHPRHSPYYIRTVRADAKDPLAVFLEAKGVPVE
           |||||||||||||||||||||||||||||||||||||||||||||||||||||||||||||||
HHblits   -AEKLGIEQSAAITTVKPSGTVSQLVDSASGIHPRHSPYYIRTVRADAKDPLAVFLEAKGVPVE
```

>aihP01D_scaffold3379*

```
BLASTx    ---------NSRVTFQTIHKLLGLTEKITNDGKQEFVNQGDFKPQIKTVRLLIVDEVSMLNDDLFHEILKYRDKIKIICM
                   |||||||||||||||||||||||||||||||||||||||||||||||||||||||||||||||||||||||
HHblits   VIKKTSGLRNSRVTFQTIHKLLGLTEKITNDGKQEFVNQGDFKPQIKTVRLLIVDEVSMLNDDLFHEILKYRDKIKIICM

BLASTx    GDPAQIPPVGKPDCIPFREELADHYRIKTLQLNQIMR----------------
          |||||||||||||||||||||||||||||||||||||
HHblits   GDPAQIPPVGKPDCIPFREELADHYRIKTLQLNQIMRQKEGNAIIDSSVIIRK
```

>aihP01D_scaffold2503*

```
BLASTx    GVDHYSKTKEYKEKFKNTMMGRFGVENPMHSEKIKKKMAERSLEKYGVECHLSLPEIQNKIKKTWESRGFXFRRNSSTEE
          ||||||||||||||||||||||||||||||||||||||||||||||||||||||||||||||||||
HHblits   GVDHYSKTKEYKEKFKNTMMGRFGVENPMHSEKIKKKMAERSLEKYGVECHLSLPEIQNKIKKTWE--------------

BLASTx    WLVSYIKTCNHKYGTDHPMQNVDYFEKCMKN

HHblits   -------------------------------
```

>aihP01D_C2868846

```
BLASTx    ----LYPDKEWSWTSISCNPNITIEYINNNPDKPWNWSTMSYNPNLTIEFIKNNLNKPW
              |||||||||||||||||||||||||||||||||||||||||||||||||||||||
HHblits   QLLELYPDKEWSWTSISCNPNITIEYINNNPDKPWNWSTMSYNPNLTIEFIKNNLNKPW
```

>aihP01D_scaffold1961*

```
BLASTx    MTTHGGNIGKIFADVGKTTLDTLEKFEIPFDEIFFGKPNADFYIDDLI
          |||||||||||||||||||||||||||||||||||||||||||||||
HHblits   MTTHGGNIGKIFADVGKTTLDTLEKFEIPFDEIFFGKPNADFYIDDL-
```

>hbvP02D_scaffold3566

```
BLASTx    -DCSKKYNLQRTQESLLEKYGTTNIMKIQGSVDKIKETNRKKYGSDFYTETDEFKKKTKETFEKKYGGHPTKLKETQDRK
          ||||||||||||||||||||||||||||||||||||||||||||||||||||||||||||||||||||||||||||||||
HHblits   DDCSKKYNLQRTQESLLEKYGTTNIMKIQGSVDKIKETNRKKYGSDFYTETDEFKKKTKETFEKKYGGHPTKLKETQDRK

BLASTx    KKTNLERYGHEHSLNNPEIKEKSRITNNLKYGGDSSMCSEEIRKKSIETNKKKRGTDWYVQSDDFKKKFKETMFLRYGVE
          ||||||||||||||||||||||||||||||||||||||||||||||||||||||||||||||||||||||||||||||||
HHblits   KKTNLERYGHEHSLNNPEIKEKSRITNNLKYGGDSSMCSEEIRKKSIETNKKKRGTDWYVQSDDFKKKFKETMFLRYGVE

BLASTx    QVMHYTPSFEKSIDTSYKKKIYIFPSGRVEKIQGYEGFGINNLLNSGYSEDDIVISNQEIEKFTGKIWYNDSENKRRKYY
          ||||||||||||||||||||||||||||||||||||||||||||||||||||||||||||||||||||||||||||||||
HHblits   QVMHYTPSFEKSIDTSYKKKIYIFPSGRVEKIQGYEGFGINNLLNSGYSEDDIVISNQEIEKFTGKIWYNDSENKRRKYY
```

```
BLASTx    PDIYIISENRIIEVKSKYT
          |||||||||||||||||||
HHblits   PDIYIISENRIIEVKSKYT
```

>hcvP02D_C2069761

```
BLASTx    LSYHPNLTMEMINIHPDKPWDWNAISSNPNLTMEFINNNPDKPWNWRPISRNSNITMQDINNNLDKPWNWGYISSNPNLT
          ||||||||||||||||||||||||||||||||||||||||||||||||||||||||||||||||||||||||||||||||
HHblits   LSYHPNLTMEMINIHPDKPWDWNAISSNPNLTMEFINNNPDKPWNWRPISRNSNITMQDINNNLDKPWNWGYISSNPNLT

BLASTx    IKMVNNNLDKPWCHRELSCHPNITIKDIINNPNELW--
          ||||||||||||||||||||||||||||||||||||
HHblits   IKMVNNNLDKPWCHRELSCHPNITIKDIINNPNELWIW
```

>hcvP02D_C2068135*

```
BLASTx    --NTLIKYFESAEPEIVCLSETKLTTDLNSDLDRLYQYKYFSHSSITKGYSGVCIYSKIKPIKEIKYNDTEGRVLCLEFK
          ||||||||||||||||||||||||||||||||||||||||||||||||||||||||||||||||||||||||||||||||
HHblits   STNTLIKYFESAEPEIVCLSETKLTTDLNSDLDRLYQYKYFSHSSITKGYSGVCIYSKIKPIKEIKYNDTEGRVLCLEFK

BLASTx    KYYLVNVYYVPNSGSDLKRLQFRLEWDNNFRIFIKRL
          |||||||||||||||||||||||||||||||||||||
HHblits   KYYLVNVYYVPNSGSDLKRLQFRLEWDNNFRIFIKRL
```

>hcvP02D_C2068561*

```
BLASTx   -MTRINLVHPSELTDQHLMAEWREIKMVPAALRRSLRTQTVQTVFKKIPREFTLNKGHVTFFYNKIDYLKERYKALTKEL-
          |||||||||||||||||||||||||||||||||||||||||||||||||||||||||||||||||||||||||||||||
HHblits  CMTRINLVHPSELTDQHLMAEWREIKMVPAALRRSLRTQTVQTVFKKIPREFTLNKGHVTFFYNKIDYLKERYKALTKELF
```

>hcvP02D_C2064825*

```
BLASTx    DFGEFDALLLGDIHKMQYLDKYNRVAYSGSLIQQNKGESVFNHGYLLWDLKNDLQSEFVEVQNDYCYLKVTIMNSDYV
             |||||||||||||||||||||||||||||||||||||||||||||||||||||||||||||||||||
HHblits   ---EFDALLLGDIHKMQYLDKYNRVAYSGSLIQQNKGESVFNHGYLLWDLKNDLQSEFVEVQNDYCYLKV--------
```

>hcvP02D_C2057559

```
BLASTx    -FDFYITDFNLIIELDGEQHFKQVSNWKSPELNLINDTNKIKLSIENNYSIIHISQDDVWNDNNNWEVKLLNCIKEYHYP
           |||||||||||||||||||||||||||||||||||||||||||||||||||||||||||||||||||||||||||||||
HHblits   RFDFYITDFNLIIELDGEQHFKQVSNWKSPELNLINDTNKIKLSIENNYSIIHISQDDVWNDNNNWEVKLLNCIKEYHYP

BLASTx    TIIFI
          |||||
HHblits   TIIFI
```

>hcvP02D_C2028534*

```
BLASTx    DGDISDWDVSRVENMAFMFDQSAFVGDIPQWDVSNVKNMEGMFSHSRFRGVIRQWDVSNVKNMEGM
          ||||||||||||||||||||||||||||||||||||||||||||||||||||||||||||||||||
HHblits   DGDISDWDVSRVENMAFMFDQSAFVGDIPQWDVSNVKNMEGMFSHSRFRGVIRQWDVSNVKNMEGM
```

>hcvP02D_C2037900*

```
BLASTx    YTNKRSRAWLKCKAEETEDLIITGAFEGEGKYVGMLGGLICDRNGVSVRCGGGFSDAQRSE
          |||||||||||||||||||||||||||||||||||||||||||||||||||||||||||||
HHblits   YTNKRSRAWLKCKAEETEDLIITGAFEGEGKYVGMLGGLICDRNGVSVRCGGGFSDAQRSE
```

>hcvP02D_C1979207**

```
BLASTx    QSLGFACYKNKKTTSWTHKGVKKHGEAWRICISGEGIEQIPTLSPRKQSLPRRQIK
          ||||||||||||||||||||||||||||||||||||||||||||||||||||||||
HHblits   QSLGFACYKNKKTTSWTHKGVKKHGEAWRICISGEGIEQIPTLSPRKQSLPRRQIK
```

>hcvP02D_C1975859

```
BLASTx    LIALLYSGEAAKWDVSNVRAAVARLKVFCGRASGPGPLEDLFRFVTKIFKGAVGR
          |||||||||||||||||||||||||||||||||||||||||||||||||||||||
HHblits   LIALLYSGEAAKWDVSNVRAAVARLKVFCGRASGPGPLEDLFRFVTKIFKGAVGR
```

>hcvP02D_C2000069**

```
BLASTx    AYDNLTIQLKSSGGRLEVVKTLFKHTNPSIDNNYAIRWASRYGYIEVEILL-
          |||||||||||||||||||||||||||||||||||||||||||||||||||
HHblits   AYDNLTIQLKSSGGRLEVVKTLFKHTNPSIDNNYAIRWASRYGYIEVEILLK
```

>hcvP02D_C2053991

```
BLASTx    -NQLQELIKHYPNKPWNWNYISENPNFNLEIINENPDKPLNWHLISNNKSIT---------------------
           |||||||||||||||||||||||||||||||||||||||||||||||||||
HHblits   INQLQELIKHYPNKPWNWNYISENPNFNLEIINENPDKPLNWHLISNNKSITVQDIENNLNKPWNWKNLSKNP
```

>hcvP02D_C1977728*

```
BLASTx    SGNITAPNLTSMVPVGSITMFAGSSAPTGWLVCDGSAFSSSAYPALYTVL
                        ||||||||||||||||||||||||||||||||||||
HHblits   --------------VGSITMFAGSSAPTGWLVCDGSAFSSSAYPALYTVL
```

>hcvP02D_C1979912*

```
BLASTx    NGVAKEQARSVLPEGCTNSRLYMSGTLRSWMHYCLLRMGNGTQKEHIVIA---
          ||||||||||||||||||||||||||||||||||||||||||||||||||
HHblits   NGVAKEQARSVLPEGCTNSRLYMSGTLRSWMHYCLLRMGNGTQKEHIVIADEI
```

>hcvP03D_C1260813

```
BLASTx    QRTQESLLEKYGTTNIMKIQGSVDKIKETNRKKYGSDFYTETDEFKKKTKETFEKKYGGHPTKLKETQDRKKKTNLERYG
          ||||||||||||||||||||||||||||||||||||||||||||||||||||||||||||||||||||||||||||||||
HHblits   QRTQESLLEKYGTTNIMKIQGSVDKIKETNRKKYGSDFYTETDEFKKKTKETFEKKYGGHPTKLKETQDRKKKTNLERYG

BLASTx    HEHSLNNPEIKEKSRITNNLKYGGDSSMCSEEIRKKSIETNKKKRG------
          ||||||||||||||||||||||||||||||||||||||||||||||
HHblits   HEHSLNNPEIKEKSRITNNLKYGGDSSMCSEEIRKKSIETNKKKRGTDWYVQ
```

>hcvP03D_scaffold3278

```
BLASTx    ----KLFHWQSHKYGQHKELDEFFDGILDLGDKLAETVMGKYGVPVLNEEQLLLKLENFTDPKSGGLKPFLEKLYRCYSE
              ||||||||||||||||||||||||||||||||||||||||||||||||||||||||||||||||||||||||||||||
HHblits   TAQIKLFHWQSHKYGQHKELDEFFDGILDLGDKLAETVMGKYGVPVLNEEQLLLKLENFTDPKSGGLKPFLEKLYRCYSE

BLASTx    EFKSLMDEKKDPELINIIDELSALVQQYKY----
          ||||||||||||||||||||||||||||||
HHblits   EFKSLMDEKKDPELINIIDELSALVQQYKYLLEL
```

>hcvP03D_C1252306

```
BLASTx    EKRKGSTRNFTEEHKIKLSKSKMADKNPMFGKQMDDEAKAHLSRITKGRKMSAEFRENVSKGKSGVKFTDEHKSNLSK--
           |||||||||||||||||||||||||||||||||||||||||||||||||||||||||||||||||||||||||||||
HHblits   -KRKGSTRNFTEEHKIKLSKSKMADKNPMFGKQMDDEAKAHLSRITKGRKMSAEFRENVSKGKSGVKFTDEHKSNLSKNH
```

>hcvP03D_scaffold1740

```
BLASTx   WNSVSCNPNITMQDIINNPDKPWNWGFISGNPNLTAQMVINNP
         |||||||||||||||||||||||||||||||||||||||||||
HHblits  WNSVSCNPNITMQDIINNPDKPWNWGFISGNPNLTAQMVINNP
```

>hcvP03D_C1243608**

```
BLASTx   RGRKLTKEWRDKISKGGIGLKRSEQTKRKISRAKKGERNPFYGKTHTAEALEKITERSKGKNNPRA
            |||||||||||||||||||||||||||||||||||||||||||||||||||||||||||||
HHblits  ---KLTKEWRDKISKGGIGLKRSEQTKRKISRAKKGERNPFYGKTHTAEALEKITERSKGKNNP--
```

>hcvP03D_C1231007*

```
BLASTx   FSKFVYYYLSGNMNILENGFKGAGLKHISKDYLENIEIPLPNLATQQRI-
          ||||||||||||||||||||||||||||||||||||||||||||||||
HHblits  -SKFVYYYLSGNMNILENGFKGAGLKHISKDYLENIEIPLPNLATQQRIA
```

>hcvP05D_C2674949*

```
BLASTx   DDAPLRDAAGYGSQETVMTLLEHGADIHARDDYALRSAAKAGNTETVKTLLEHGANIHAQHGEALKLAAENGH
          ||||||||||||||||||||||||||||||||||||||||||||||||||||||||||||||||||||||||
HHblits  -DAPLRDAAGYGSQETVMTLLEHGADIHARDDYALRSAAKAGNTETVKTLLEHGANIHAQHGEALKLAAENGH
```

>hcvP05D_C2666909

```
BLASTx   LSSNSNLTLSFILNNQDRDWDWDYVSMNTNLKIQDIINNIDKPWDWSELSLNE----
         |||||||||||||||||||||||||||||||||||||||||||||||||||||
HHblits  LSSNSNLTLSFILNNQDRDWDWDYVSMNTNLKIQDIINNIDKPWDWSELSLNEFMIY
```

>norP01D_scaffold1274**

```
BLASTx   IYMITSPSGRVYVGQSVDIGRRFKEYRRSGSARKQRRLAASFAKYGVDSHVFCVVQECAENVLSDMERQWQERLMVCGTG
         ||||||||||||||||||||||||||||||||||||||||||||||||||||||||||||||||||||||||||||||||
HHblits  IYMITSPSGRVYVGQSVDIGRRFKEYRRSGSARKQRRLAASFAKYGVDSHVFCVVQECAENVLSDMERQWQERLMVCGTG

BLASTx   GLNCRLVGTGDKTGRFSESSRQRMSEKQRGEGNPNFGKRGVETSCFGRKRTDDERKSISDFQ------------------
         ||||||||||||||||||||||||||||||||||||||||||||||||||||||||||||||
HHblits  GLNCRLVGTGDKTGRFSESSRQRMSEKQRGEGNPNFGKRGVETSCFGRKRTDDERKSISDFQRQRGQIILQIDPASGLIV

BLASTx   --------------------------------------

HHblits  RKARSWEYVADGFSQGNISSCCTGRLKTHKGFQFQYEA
```

>norP01D_C2719632

```
BLASTx   HPTKLKETQDRKKKTNLERYGHEHSLNNPEIKEKSRITNNLKYGGDSSMCSEEIRKKSIETNKKKRGTDWYVQSDDFKKK
              |||||||||||||||||||||||||||||||||||||||||||||||||||||||||||||||||||||||||||
HHblits  -----KETQDRKKKTNLERYGHEHSLNNPEIKEKSRITNNLKYGGDSSMCSEEIRKKSIETNKKKRGTDWYVQSDDFKKK

BLASTx   FHE

HHblits  ---
```

>norP01D_C2726684*

```
BLASTx    --------------------------------------------------------------------------------

HHblits   LASWTNPNARAQMALTAIESFKGAKNKHPMTLCKCDCGGERLVRTTRFRLGLVSSCAACARREAAARGLTRSLPVDVAMN


BLASTx    ----------ARRRGLVFDLNEEQVTALIRSACLYCGAPADPTNGIDRRDNSEGYTAANAVPCCSMCNYAKRDLTDQQFL
                    ||||||||||||||||||||||||||||||||||||||||||||||||||||||||||||||||||||||
HHblits   REIRGVYLGNARRRGLVFDLNEEQVTALIRSACLYCGAPADPTNGIDRRDNSEGYTAANAVPCCSMCNYAKRDLTDQQFL


BLASTx    EWAKRIHDH
          ||||||||
HHblits   EWAKRIHD-
```

>norP01D_C2716188*

```
BLASTx    KEIYKNTMNEKYGVDHYSKTKEYKEKFKNTMMGRFGVENPMHSEKIKKKMAERSLEKYGVECHLSLPEIQNKIK
           |||||||||||||||||||||||||||||||||||||||||||||||||||||||||||||||||||||
HHblits   -EIYKNTMNEKYGVDHYSKTKEYKEKFKNTMMGRFGVENPMHSEKIKKKMAERSLEKYGVECHLSLPEIQ----
```

>nshP01D_C2726334*

```
BLASTx    -GDYSTVEPIVKNIEYLRFLKESGHHIIIYTARKMTTHGGNIGKIFADVGKTTLDTLEKFEIPFDEIFFGKPNTDFYIDD
           ||||||||||||||||||||||||||||||||||||||||||||||||||||||||||||||||||||||||||||
HHblits   KGDYSTVEPIVKNIEYLRFLKESGHHIIIYTARKMTTHGGNIGKIFADVGKTTLDTLEKFEIPFDEIFFGKPNTDFY---


BLASTx    LAINSNHEISKLTGFFDCNI

HHblits   --------------------
```

>nshP01D_C2719700**

```
BLASTx    NPEIKEKSRITNNLKYGGDSSMCSEEIRKKSIETNKKKRGTDWYVQSDDFKKKFKETMFLRYGVEQVMHYTPSFEKSIDT
           |||||||||||||||||||||||||||||||||||||||||||||||||||||||||||||||||||||||||||||||
HHblits   -PEIKEKSRITNNLKYGGDSSMCSEEIRKKSIETNKKKRGTDWYVQSDDFKKKFKETMFLRYGVEQVMHYTPSFEKSIDT


BLASTx    SYKKKIYIFP
          ||||||||||
HHblits   SYKKKIYIFP
```

>nshP01D_scaffold4755**

```
BLASTx    YKRFSKKIGRQFSITFLDFAEIVKNPXAGDVKYLTTLHGKSTITDIFNGIDRKDNSIGYVKENCLPCCKMCNYMKGSYGH
                                           |||||||||||||||||||||||||||||||||||||||||||||||
HHblits    --------------------------------LTTLHGKSTITDIFNGIDRKDNSIGYVKENCLPCCKMCNYMKGSYGH


BLASTx    EEFISKCHLISSNFNKI-----
          |||||||||||||||||
HHblits   EEFISKCHLISSNFNKIYVKLP
```

>nshP01D_C2724894**

```
BLASTx    EKIGLSVRGRKLTKEWRDKISKGGIGLKRSEQTKRKISRAKKGERNPFYGKTHTAEALEKITERSKGKNNPRAIVVQAVN
          ||||||||||||||||||||||||||||||||||||||||||||||||||||||||||||||||||||||||||||||||
HHblits   EKIGLSVRGRKLTKEWRDKISKGGIGLKRSEQTKRKISRAKKGERNPFYGKTHTAEALEKITERSKGKNNPRAIVVQAVN


BLASTx    KDG---
          |||
HHblits   KDGMIL
```

>nshP01D_C2709265

```
BLASTx    NIVSPREVFKSSGKYYLFDCNICSHEFKIQLSNITRGMWCNFCSSTQLCNDDCFICFNKSFASSDKVKYWSEKNIVSP
          ||||||||||||||||||||||||||||||||||||||||||||||||||||||||||||||||||||||||||||||
HHblits   NIVSPREVFKSSGKYYLFDCNICSHEFKIQLSNITRGMWCNFCSSTQLCNDDCFICFNKSFASSDKVKYWSEKNIVSP
```

>nshP01D_C2709611

```
BLASTx    KNGKRYYETNKEQIFEYQKQYSQNNKEKIAKRAKKWREENKECILEKKKQYYQNNKEHHAESVQQWREENKELVLKYSKK
                    |||||||||||||||||||||||||||||||||||||||||||||||||||||||||||||||||||
HHblits   ----------KEQIFEYQKQYSQNNKEKIAKRAKKWREENKECILEKKKQYYQNNKEHHAESVQQWREENKELVLKY---
```

>nshP01D_scaffold737

```
BLASTx    --YGVDHYSKTKEYKEKFKNTMMGRFGVENPMHSEKIKKKMAERSLEKYGVECHLSLPEIQNKIKKT-------------
            |||||||||||||||||||||||||||||||||||||||||||||||||||||||||||||||||
HHblits   EKYGVDHYSKTKEYKEKFKNTMMGRFGVENPMHSEKIKKKMAERSLEKYGVECHLSLPEIQNKIKKTWESRGFKRNSSTE

BLASTx    -----------------

HHblits   EWLVSYIKTSNHKYGTD
```

>nshP01D_C2677940*

```
BLASTx    WGALYESKSGERGIFNRQAAKLKSEANGRRLSDVDFGTNPCGEIILRPFGFCNLSEVVV
          |||||||||||||||||||||||||||||||||||||||||||||||||||||||||||
HHblits   WGALYESKSGERGIFNRQAAKLKSEANGRRLSDVDFGTNPCGEIILRPFGFCNLSEVVV
```

>nshP01D_C2641591

```
BLASTx    NNPDKPWDWKWLSMNPNLTMQFINDNLDQHWSWNWISANSGITIQDVINNPDKPW
           ||||||||||||||||||||||||||||||||||||||||||||||||||||||
HHblits   -NPDKPWDWKWLSMNPNLTMQFINDNLDQHWSWNWISANSGITIQDVINNPDKPW
```

>nshP01D_C2667438

```
BLASTx    -NSSAIHLLEAYPEKIDWVYLSTNPNAIHLLEANPEKIDWVYLSMNPNAIHLLEAN-
           |||||||||||||||||||||||||||||||||||||||||||||||||||||||
HHblits   RNSSAIHLLEAYPEKIDWVYLSTNPNAIHLLEANPEKIDWVYLSMNPNAIHLLEANP
```

>nshP01D_C2645388*

```
BLASTx    FAEDKMETFTKAVGTPIYMAPEVMTTKNYSKEADIYSFGILLWVIITQKEPY
                    ||||||||||||||||||||||||||||||||||||||||||
HHblits   ----------KAVGTPIYMAPEVMTTKNYSKEADIYSFGILLWVIITQKEPY
```

>nshP01D_C2611137*

```
BLASTx    -PVHFAASNGHVECIRILVKCGALIDTLDENGLLPSYYAACDKHVECLQLL
           ||||||||||||||||||||||||||||||||||||||||||||||||||
HHblits   YPVHFAASNGHVECIRILVKCGALIDTLDENGLLPSYYAACDKHVECLQLL
```
